# A Review of Antibacterial Agents in Endodontic Treatment

**Published:** 2014-07-05

**Authors:** Saeed Rahimi, Maryam Janani, Mehrdad Lotfi, Shahriar Shahi, Amirala Aghbali, Mahdi Vahid Pakdel, Amin Salem Milani, Negin Ghasemi

**Affiliations:** a Dental and Periodontal Research Center, Dental School, Tabriz University of Medical Sciences, Tabriz, Iran; b Department of Endodontics, Dental school, Tabriz University of Medical Sciences, Tabriz, Iran; c Research Center for Pharmaceutical Nanotechnology and Department of Endodontics, Tabriz University of Medical Sciences, Tabriz, Iran; d Department of Prosthodontics, Dental school, Tabriz University of Medical Sciences, Tabriz, Iran

**Keywords:** Antibacterial Agents, Calcium Hydroxide, Chlorhexidine, Lasers, MTAD, Ozone, Root Canal Irrigation, Root Canal Treatment, Sodium Hypochlorite

## Abstract

Microorganisms play a major role in initiation and perpetuation of pulpal and periapical diseases. Therefore, elimination of the microorganisms present in the root canal system is the fundamental objective of endodontic treatment. The use of mechanical debridement, chemical irrigation or other antimicrobial protocols and intra-canal medicaments are critical to attain this goal. The aim of this article was to review the antimicrobial agents and their properties in endodontics.

## Introduction

Microorganisms are the main causes of pulpal and periapical diseases. Curing the existing “apical periodontitis” or its prevention by disinfection of the root canal system (RCS) and prevention of its re-infection are the primary goals of endodontic treatment [1-4]. Elimination of microorganisms from the infected RCS and rendering these spaces bacteria free, is hard, if not impossible. The morphology of the RCS is very complicated [5, 6] and mechanical preparation alone is not sufficient to disinfect accessory canals, anastomoses and fins [4, 7, 8]. Numerous approaches have been suggested for reducing the number of microorganisms from the root canal system, including the use of various instrumentation techniques, irrigation regiments and inter-appointment intracanal medicaments. In the literature, mechanical instrumentation alone is not considered enough for disinfection of the RCS which is not surprising considering the complex anatomy of the pulp space [9]. Besides using aseptic principles such as rubber dam isolation and precise mechanical instrumentation and considering the fact that most of the root canal filling and sealing materials have limited antimicrobial effect, root canal irrigants are the key factor in eradication of microbes from the RCS [10-13].

To increase the efficacy of mechanical preparation and bacterial removal, instrumentation must be supplemented with efficient intracanal irrigants. Irrigation is defined as washing out a body cavity or wound with water or medical fluid. Thus the objective of irrigation is both mechanical and biologic. While the former is due to flushing effect, the latter is related to the antimicrobial properties of the irrigant [14]. The ideal irrigant should be germicide and fungicide, non-toxic, nonirritating for host tissues, not interfering with tissue repair, stable in solution, have prolonged antimicrobial effect and is preferred to be relatively inexpensive [15, 16].

Agents for chemical treatment of the RCS can be divided into several phases, namely irrigants, rinses, and inter-appointment medicaments, the properties of which are being discussed in the present review, besides discussing the modern approaches in disinfection of the RCS [4].

***Sodium hypochlorite***

First introduced as bleaching agents, hypochlorite solutions gained wide acceptance as disinfectants by the end of the 19^th^ century based on the controlled laboratory studies by Koch and Pasteur [17]. In World War I, Henry Drysdale Dakin (HD Dakin) and Alexis Carrel extended the use of a buffered 0.5% solution of sodium hypochlorite (NaOCl) to the irrigation of infected wounds, based on Dakin’s meticulous studies on the efficacy of different solutions on infected necrotic tissues [18].

Besides their wide spectrum and nonspecific killing efficacy on all microbes, hypochlorite preparations are both sporocidal and virucidal and also have much stronger dissolving effect on necrotic rather than vital tissues [19, 20]. These features prompted the use of aqueous NaOCl as the main irrigant in endodontics in early 1920s [21, 22]. Because of the complexity of irregular RCS, sufficient instrumentation may be impossible; therefore, NaOCl can improve root canal cleaning [23-25].

In the endodontic field, NaOCl shows a broad spectrum antimicrobial activity against difficult-to-eradicate microorganisms and biofilms of species such as *Enterococcus*, *Actinomyces* and *Candida*. Furthermore, NaOCl solutions are non-expensive, easily available, and have a long shelf life [26-28]. Other chlorine-releasing compounds have been advocated in endodontics, such as chloramine-T and sodium dichloroisocyanurate, which however never gained wide acceptance in endodontics, and appear to be less effective than hypochlorite at comparable concentrations [29].

There has been controversy over the most effective and meanwhile non-toxic concentration of hypochlorite solutions to be used in endodontics. As Dakin’s original solution (0.5% NaOCl) was designed to treat open wounds, it was surmised that in the confined area of a RCS, higher concentrations should be used to be more efficient than Dakin’s solution [21]. The antibacterial effectiveness and tissue-dissolution capacity of aqueous hypochlorite is a function of its concentration, but so is its toxicity [30, 31]. However, severe irritations have been reported when 5.25% solutions were inadvertently forced into the periapical tissues during irrigation [32]. Furthermore, a 5.25% solution significantly decreases the elastic modulus and flexural strength of human dentin compared to physiologic saline, while a 0.5% solution does not [33]. This is most likely due to the proteolytic action of fully concentrated hypochlorite on the collagen matrix of dentin. Moreover compared to the 0.5% solution, the reduction of intracanal microbiota is not any greater when 5.25% NaOCl is used [34]. From *in vitro* observations, it appears that a 1% NaOCl solution should suffice to dissolve the entire pulp tissue during an endodontic treatment session [35]. Hence, based on the currently available evidence, there is no rationale for using hypochlorite solutions at concentrations over than 1% wt/vol. The same concentration of NaOCl is also used for disinfection of gutta-percha cones [36]. At body temperature, reactive chlorine in aqueous solution can take two forms: hypochlorite (OCl) and hypochlorous acid (HOCl) in pH values above or below 7.6, respectively. Both forms are extremely reactive oxidizing agents [17]. Pure hypochlorite solutions as they are used in endodontics have a pH of 12, and thus the entire available chlorine is in the form of OCl. However, at identical levels of available chlorine, hypochlorous acid is more bactericidal than hypochlorite [37].

One way to increase the efficacy of hypochlorite solutions could be lowering the pH, *i.e.* by buffering the solution with 1% bicarbonate [37]. It has also been surmised that such solutions would be less toxic to vital tissues than their non-buffered counterparts [38]. However, buffering hypochlorite with bicarbonate renders the solution unstable with a decrease in its shelf life to less than 1 week. Depending on the amount of the bicarbonate in the mixture and therefore the pH value, the antimicrobial efficacy of a fresh bicarbonate-buffered solution is only slightly higher if not similar, than that of a non-buffered solution [39]. Another approach to improve the effectiveness of hypochlorite irrigants in the RCS could be to increase the temperature of low-concentration NaOCl solution. This improves its immediate tissue-dissolution capacity [40]. Furthermore, heated hypochlorite solutions remove organic debris from dentin shavings more efficiently. Increasing the temperature of NaOCl by 5 degrees, doubles its activity [41].

***Chlorhexidine***

Chlorhexidine (CHX) is a strong basic solution and is most stable in the form of salt. The original salts were CHX acetate and hydrochloride, both of which are poorly soluble in water [42, 43]. Hence, they were replaced by CHX digluconate [44]. It has a cationic molecular component that attaches to negatively charged cell membrane and causes cell lysis. CHX is a potent antiseptic, which is used as a mouth rinse and endodontic irrigant. The later application is based on its substantivity and long-lasting antimicrobial effect which arise from its tendency to bind to hydroxyapatite [45]. Aqueous solutions of 0.1 to 0.2% concentrations are recommended for that purpose, while 2% is the concentration of root canal irrigating solutions usually found in the endodontic literature [30]. It is commonly held that CHX would be less caustic than NaOCl [30], however a 2% solution is irritating to the skin [42]. As with NaOCl, heating CHX of lesser concentrations could increase its local efficacy in the root canal system while keeping the systemic toxicity low [46]. Despite its usefulness as a final irrigant, CHX cannot be advocated as the main irrigant in standard endodontic cases, because of many issues: *i*) CHX is unable to dissolve necrotic tissue remnants [47], and *ii*) CHX is less effective on gram-negative than on gram-positive bacteria [48]. Moreover, the most important disadvantage of CHX is its inability to dissolve remnants of necrotic tissues and chemically clean the RCS [49]. In a randomized clinical trial comparing the efficiency of either 2.5% NaOCl or 0.2% CHX irrigation in reduction of intracanal microbiota, it was found that NaOCl was significantly more efficient than CHX in obtaining negative cultures [23, 50]. Schafer and Bossmann reported that 2% CHX gluconate was significantly more effective against *E. faecalis *than calcium hydroxide (CH) used alone, or a mixture of the two [51]. This was also confirmed by Lin *et al. *[52]. Although in a study by Evans *et al. *[53] on bovine dentine, 2% CHX with CH was shown to be more effective than CH mixed with water. Waltimo *et al. *reported that 0.5% CHX-acetate was more effective in killing *Candida albicans (C. albicans) *than saturated CH, while CH combined with CHX was more effective than CH used alone [54]. Another study evaluated the effectiveness of 2% CHX solution mixed with CH against *C. albicans *and found that a combination of the two was beneficial [55].

CHX has been used in endodontics and proposed as both an irrigant and an intracanal medicament. It is active against a wide range of microorganisms, such as gram-positive and gram-negative bacteria including *Enterococcus faecalis *(*E. faecalis)*, yeasts and fungi. When used as an intracanal medicament, CHX is more effective than CH against *E. faecalis *infection in dentinal tubules [56-58]. In fact, the antimicrobial activity of CHX is reduced when combined with other substances, including CH and CH plus zinc oxide, among others [56, 59-61]. For endodontic purposes, CHX can be used in a liquid or in a gel presentation. Ferraz *et al*. showed that 2% CHX gel has several advantages over 2% CHX solution, in spite of having similar antimicrobial, substantivity and biocompatibility properties [62, 63].

The use of CHX gel as an intracanal medicament is recommended for a short period of time (3-5 days), particularly in those cases where the canals were fully instrumented but could not be filled due to the lack of time. It is also recommended in cases of exudation (unpublished data), as it retains its antimicrobial activity in the presence of blood and other organic matters [42].

***Iodine potassium iodine***

Iodide potassium iodine (IKI) is a traditional root canal disinfectant with wide-spectrum antimicrobial activity. It is used in different concentrations ranging from 2% to 5%. Iodine, as the oxidizing agent of this substance, reacts with free sulfhydryl groups of bacterial enzymes cleaving the disulfide bonds [64]. It was manifested that CH-resistant microorganisms could be eradicated with a combination of IKI and CHX [65, 66]. It shows relatively low toxicity in experiments using tissue cultures. An obvious disadvantage of iodine is a possible allergic reaction in some patients, which can be the cause for inter-appointment pain [67].

***MTAD***

A Mixture of Tetracycline, Acid and Detergent, labeled as Biopure MTAD (Dentsply, Tulsa Dental, Tulsa, OK, USA), was introduced as an antibacterial root canal cleanser [44, 68]. This biocompatible intracanal irrigant is commercially available as a two-component mix [69]. One of the characteristics of this solution is the high binding affinity of the doxycycline component to dentin [70]. In this irrigant, doxycycline hyclate is used instead of its free base, doxycycline monohydrate, to increase the water solubility of this broad-spectrum antibiotic [70]. MTAD has been reported to be able to remove the smear layer due to the action of citric acid [71, 72], effectively eliminate microorganisms that are resistant to conventional endodontic irrigants/medications [73] and provide sustained antimicrobial activity [73-75]. MTAD was compared with commonly used irrigants and medications. The results showed MTAD to be less cytotoxic than eugenol, 3% H_2_O_2_, CH paste, 5.25% NaOCl, 0.12% CHX gluconate, and 17% EDTA. MTAD is more cytotoxic than NaOCl at 2.63%, 1.31%, and 0.66% concentrations.

Tetraclean (Ogna Laboratori, Farmaceutici, Milano, Italy) is another combination product similar to MTAD. The two irrigants differ in the concentration of antibiotics (doxycycline 150 mg/5ml for MTAD and 50 mg/5ml for Tetraclean) and the type of detergent (Tween 80 for MTAD). Mohammadi *et al*. showed that the substantivity of Tetraclean was significantly higher than that of MTAD [76].

***Calcium hydroxide***

Calcium hydroxide [Ca(OH)_2_] (CH) is a white odorless powder that was originally introduced to the field of endodontics by Herman as a direct pulp-capping agent [4]. It is generally believed that the number of residual bacteria are responsible for endodontic failures [77]. It can be controlled by placing an inter-appointment medicament within the prepared canal [78-80], and CH, is the most commonly used inter-appointment dressing which at least requires a period of 7 days for efficient disinfection [81]. Antimicrobial activity of CH is related to the release of hydroxyl ions (OH^-^) in an aqueous environment which is probably due to the damage to the bacterial cytoplasmic membrane; protein denaturation; and damage to their DNA [4]. However, some microorganisms such as *E. faecalis *[82] and *C. albicans *[54] are resistant to CH. Therefore, alternative intracanal medications have been sought to improve the eradication of bacteria before obturation. CHX gluconate is shown to be effective against some CH-resistant strains [83]. Recent studies have suggested that considering this synergistic activity, CHX could be used in combination with CH to improve the antimicrobial efficacy [57]. The high pH of CH (*i.e.* 12.5) alters the biologic properties of bacterial lipopolysacharide (LPS) present in the cell walls of gram-negative species and also inactivates the membrane transport mechanisms which has a role in killing the microorganism [84]. However, as stated above, *E. faecalis *has been reported to be resistant to this effect as a result of its ability to penetrate the dentinal tubules and adapt to changes in the environment [82].

***Laser irradiation and photodynamic therapy***

Recently, novel approaches in disinfection of RCS have been proposed that include the use of high-power lasers [85] as well as photodynamic therapy (PDT) [86]. High-power lasers function by dose-dependent heat generation, and apart from bacterial killing properties, if incorrect parameters are used they have the potential to cause collateral damage such as char dentine, ankylosis of the roots, cementum melting, root resorption and periradicular necrosis [87, 88]. Since the introduction of laser to endodontics in 1971, several lasers were used for eliminating the bacteria from RCS. The erbium, chromium: yttrium-scandium-gallium-garnet laser (Er, Cr: YSGG), has the highest absorption in water and high affinity to hydroxyapatite, which make it suitable for root canal therapy [89, 90]. Lasers have the ability to clean and effectively disinfect the RCS, from the highly resistant species such as *E. faecalis* [91]. The effect of neodymium: yttrium-aluminum-garnet laser (Nd: YAG) on *E. faecalis* biofilm is less than that of 1% NaOCl solution. A combination of laser and NaOCl results in complete elimination of *E. faecalis* biofilms [24].

PDT is a new antimicrobial strategy that involves the combination of a nontoxic photosensitizer (PS) and a light source [92]. The excited PS reacts with molecular oxygen to produce highly reactive oxygen species, which induces injury and death of microorganisms [93]. It has been established that PS, which has a high cationic charge, can rapidly bind and penetrate the bacterial cells and therefore, shows a high degree of selectivity for killing microorganisms compared to host mammalian cells [94]. PDT seems a promising approach in eradication of oral pathogenic bacteria [95] that can cause diseases such as periodontitis, peri-implantitis and caries [85]. When conventional endodontic therapy was followed by PDT, there was significantly more bacterial killing and less bacterial growth than endodontic therapy alone [96]. Laser energy is being considered useful in treating diseases of the RCS and periradicular regions.

***Ozone***

Oxygen/ozone therapy has a long history of research and clinical/therapeutic applications on humans. The first medical application was in 1870 when Lender purified blood in test tubes [11, 97, 98].

Ozone (O_3_), is a triatomic molecule of oxygen with a molecular weight of 47.98 g/mol. Thermodynamically, this molecule is highly instable and decomposes to pure oxygen (O_2_) with a short half-life in particular temperature and pressure conditions [99].

In the clinical setting, an oxygen/ozone generator simulates lightning via an electrical discharge field [100]. Ozone gas has a high oxidation potential and it is 1.5 times more effective than chloride when used as an antimicrobial agent against bacteria, viruses, fungi, and protozoa. It also has the ability to increase blood circulation and upregulate the immune response [11].

Ozone is applied to oral tissues in the following forms: ozonated water, ozonated olive oil, and oxygen/ozone gas. Ozonated water and olive oil as the ideal delivery systems have the capacity to entrap and then release oxygen/ozone. These forms of application are used individually or in combination to treat dental disease [101]. In clinical endodontic practice, ozone has been used in a gaseous form (4.2×10 6 µg m-3 HealOzone; KaVo, Biberach, Germany) [11]. Most studies on the applications of ozone in endodontics have focused on its antimicrobial activity. Nagayoshi *et al. *found that ozonated water (0.5-4 mg/L) was highly effective in killing both gram-positive and -negative microorganisms [102]. Gram-negative bacteria, such as *Porphyromonas endodontalis *(*P. endodontalis*) and *Porphyromonas gingivalis *(*P. gingivalis*), were substantially more sensitive to ozonated water than gram-positive oral *streptococci* and *C. albicans *in pure culture [67]. The antibacterial activity of gaseous ozone was shown to be greater than KTP laser and less than NaOCl [103], and ozone gas delivered into irrigating fluids in the root canal may be useful as an adjunct for endodontic disinfection [104]. Ozone inhalation can be toxic to the pulmonary system and other organs. Because of high oxidative power of ozone, all materials that come in contact with the gas must be ozone resistant, such as glass, silicon, and Teflon [11].

Ozone improves wound healing, assists in treating root surface caries and can be used against endodontic microbiota. Furthermore, it seems that ozone does not have significant adverse effect on dentin bonding. In spite of infrequency of side effects, ozone therapy may cause serious medical complications if incorrectly used. Therefore, care must be taken in handling ozone.

## Conclusion

Root canal irrigants play an important role in eradication of microbes from the root canal system. To increase the efficacy of mechanical preparation and bacterial removal, instrumentation must be supplemented with active irrigating solutions, medicaments and/or new techniques.
